# Lower extremity kinematic analysis in male athletes with unilateral anterior cruciate reconstruction in a jump-landing task and its association with return to sport criteria

**DOI:** 10.1186/s12891-019-2893-5

**Published:** 2019-10-27

**Authors:** Sadegh Norouzi, Fateme Esfandiarpour, Sina Mehdizadeh, Nasim Kiani Yousefzadeh, Mohamad Parnianpour

**Affiliations:** 1 Musculoskeletal Rehabilitation Research Center, Ahvaz Jundishapur University of Medical Sciences, Ahvaz, Iran; 2 Department of Family Medicine, University of Alberta, Edmonton, Canada; 3 Toronto Rehabilitation Institute, University of Health Network, Toronto, Canada; 4 Department of Mechanical Engineering, Sharif University of Technology, Tehran, Iran

**Keywords:** Anterior cruciate ligament, Landing kinematics, Return to sport, Clinical decision-making, soccer

## Abstract

**Background:**

Return to sport (RTS) criteria are widely being used to identify anterior cruciate ligament reconstructed (ACLR) athletes ready to return to sportive activity and reduce risk of ACL re-injury. However, studies show a high rate of ACL re-injury in athletes who passed RTS criteria. This indicates that the current RTS criteria might not be sufficient to determine return to sport time in ACLR athletes. Previous studies have reported a close association between altered lower limb kinematics and ACL re-injury. However, it is not clear how lower extremity kinematics differs between ACLR athletes who passed the RTS-criteria and who failed. This study compared lower extremity kinematics in a jump-landing task between ACLR athletes who passed the RTS criteria (Limb symmetry in hop tests, quadriceps strength and questionnaires) to those who failed and to the healthy individuals.

**Methods:**

Participants were 27 male football players with unilateral ACLR including 14 who passed -RTS criteria and 13 failed, and 15 healthy football players. A 3D motion capture system recorded participants’ lower extremity motion while performing 10 trials of a bilateral jump-landing task. Hip, knee and ankle angular motion were examined at initial contact. Two-way mixed analysis of variances (2 limbs × 3 groups) and Bonferroni post-hoc tests were performed to compare the joint angles between the limbs and groups.

**Results:**

lower hip abduction angle was found in the failed (involved limb 4.1 ° ± 4.2) and passed RTS (involved limb 6.8° ± 3.3) groups compared to the healthy group (non-dominant limb 10.7° ± 3.7). Ankle inversion in the failed RTS (0.4° ± 4.9) group was significantly lower than both passed RTS (4.8° ± 4.8, *p* = 0.05) and healthy (8.2° ± 8.1, *p* < 0.001) groups. There were no significant differences between the groups in knee kinematics.

**Conclusions:**

Our findings indicate reduced hip abduction during initial contact phase of landing in athletes returned to sport. Reduced hip abduction during the complex multiplanar movement of jump-landing is a risk factor for ACL re-injury. Current RTS criteria may not be sufficient to identify ACLR athletes at high risk of re-injury. The kinematic analysis in conjunction with current RTS criteria can provide additional insight into the return to sport decision making.

## Background

Anterior Cruciate Ligament reconstruction (ACLR) followed by rehabilitation is the ‘gold standard’ protocol following an ACL injury in athletes, with the ultimate goal to return to sport [1]. Return to sport (RTS) criteria has been developed to return athletes as fast as possible to their pre-injury performance level while reducing the risk of re-injury [2–4]. RTS is based on a decision-making process including multidimensional aspects [3]. Assessing the level of functional/neuromuscular restoration by using quadriceps strength test, single-legged hop tests, and self-report questionnaires [5] is the most important aspect of the RTS process.

Despite ongoing progress in surgery and rehabilitation protocols for ACLR, recent studies show that athletes are still at a high risk of ACL re-injury post-surgery [6, 7]. It has been reported that ACL re-injury rates range from 22 to 30% in both affected and contralateral knees and up to 10% in the ipsilateral knee within a 10-year follow-up period [7]. Bien et al. (2015) suggested that the high rate of ACL re-injury in athletes could indicate flaws in the return to sport criteria [2].

The underlying causes for ACL re-injury are multifactorial and not limited to muscle strength deficit and/or functional performance as considered in RTS [6]. Previous studies consistently reported an association between lower limb kinematics especially during multiplanar and complicated activities such as landing and ACL injury/re-injury [8–10]. Moreover, the results of prospective studies by Paterno et al. (2010), and in Leppänen et al. (2017) indicated that altered landing biomechanics is an important predictive factor of ACL injury/re-injury [8, 11]. Based on Kyritsis et al. (2016) study, current RTS criteria have an acceptable predictability to identify athletes at risk of re-injury [12]. Despite this, the rate of ACL re-injury remain high [2]. This might highlights importance of kinematic analysis together with current RTS to provide more detailed information to return to sport decision making. It is not clear whether a good performance in muscle symmetry and functional test as measured in RTS is also indicative of a safe lower extremity kinematic pattern.

Despite the close association between altered lower limb kinematics and ACL re-injury [8, 13], there is still a lack of knowledge regarding kinematic differences between athletes who passed the RTS-criteria and who failed. A recent study by Chang et al. (2019) investigating knee kinematic differences in female athletes who pass or fail RTS criteria pass and healthy females found no significant difference between in knee kinematics between the groups [14]. However, there is still no related report regarding kinematic differences in male athletes and in the hip and ankle kinematic as contributing factors to ACL re-injury in ACLR athlete [15]. Investigation of lower extremity landing kinematic differences in these two groups the of athletes and to their healthy counterparts could reveal the underlying cause of ACL re-injury in passed-RTS athletes and will serve as the first step towards a more accurate decision making on return to sport readiness of ACLR athletes.

The present study thus aimed to analyze hip, knee and ankle 3D joint kinematics during the initial contact phase of landing task, i.e. minimum vertical velocity of ankle segment [16], in three groups of athletes: passed-RTS, failed-RTS, and healthy individuals. We hypothesize that the lower limb landing kinematics is different between the three groups and this difference is not revealed in the RTS criteria.

## Methods

### Participants

Twenty-seven athletes with ACLR (23.8 ± 3.3 years) and 15 healthy athletes with no history of any lower extremity injury (24.6 ± 3.1 years) participated in this cross-sectional study. Out of 27 participants with unilateral ACLR, 14 passed the RTS criteria (passed-RTS group), while 13 did not (failed-RTS group). Table 1 presents the characteristics of the participants of the three groups. All Participants were male football players, recruited from the local sport physiotherapy clinics and football clubs over a period of 6 months. Inclusion criteria were: age 18 to 40 years, unilateral ACL reconstruction at least 6 months before the study beginning [5, 17], ability to perform regular jumping and pivoting activities (≥50 h per year) before the injury [18]. The exclusion criteria were a meniscal tear of greater than grade I [19], osteochondral defect, a complete tear of other knee ligaments, a history of other lower extremity joint surgery and neuromuscular disorders in the hip, knee, ankle and lower back that limits the ability of athletes to perform sports activities. Participants in the control group had no history of lower limb and back injury or surgeries. The ACLR participants completed a progressive rehabilitation treatment supervised by licensed sports physiotherapists, and had full knee ROM, knee joint effusion of grade trace or zero based on the stroke test [20] and ability to perform the single-hop test without pain. A sport physiotherapist screened participant for the inclusion/exclusion criteria. All participants read and signed an informed consent approved by the university ethics committee (ethics approval number: IR.AJUMS.REC.1396.579).

**Table 1:** Participant characteristics, mean (SD)

	Failed-RTS	Passed-RTS	Healthy	F	*Sig.*
Age (year)	24.3 (3.8)	23.1 (2.5)	24.6 (3.1)	0.747	0.412
Height (cm)	176.3 (6.3)	179.5 (6.2)	174.6 (4.6)	2.501	0.094
Weight (kg)	72.1 (9.1)	73.9 (8.1)	69.1 (5.2)	1.405	0.257
Time since Injury (months)	7.5 (1.5)	7.7 (1.3)	–	0.135	0.734
Tegner Score before injury	8.1 (1.1)	8.6 (0.7)	8.4 (1.1)	1.427	0.252
Tegner Score after injury	6.8 (0.75)	8.1 (1.1)	–	**11.982**	**0.002**

*Abbreviations*: *RTS* Return to Sport, *F* F ratio, *Sig* Significance set at *p* < 0.05

### Study procedure

Participants underwent RTS and kinematic assessments in two separate testing sessions. In the first session, their demographic data including age, weight, height, and Tegner score (before the injury and 6 months after ACLR) [21] were recorded. They were also asked to fill the Knee Outcome Survey–Activities of Daily Living Scale (KOS-ADL) and the Global Knee Rating Scale questionnaires for each limb separately and without any specific cutoff [22]. An experienced sports physiotherapist performed the RTS testing. Participants were allocated to the failed and passed RTS group based on the outcomes of four commonly accepted RTS criteria including maximal isometric quadriceps strength, four functional single-legged hop tests (single, triple, crossed and 6-m-time-to-hop), the KOS-ADL and the Global Knee Rating Scale questionnaires. These particular tests are considered important to estimate the functional/neuromuscular status of athlete who want return to their pre-injury level of activities [3]. Quadriceps strength test was performed using a hand held dynamometer according to the standard belt-stabilized method [23]. For the hop tests for distance, athletes stood on one leg with their toes behind the starting line, hopped forward as far as possible and landed on the same leg. For the single and triple hop tests, athletes performed a single hop, and three consecutive hops, respectively. For the crossed hop test, they performed three consecutive hops while crossing the midline with each hop. The distance of hop was recorded for each test in centimeters (cm). For the 6-m hop test, they hopped on one leg as quickly as possible over a distance of 6 m. The tests were performed in random order and for both legs to calculate the limb symmetry index (LSI) ($\frac{\mathrm{involved}}{\mathrm{uninvolved}}$) [24]. To pass the RTS criteria, each participant had to gain a minimum LSI of 0.90 on all RTS criteria. All RTS tests were performed at least 6 months after ACL reconstruction [5].

### Kinematic evaluation

A 7-camera (240 Hz) motion analysis system (Qualysis, Gothenburg, Sweden) was used to capture the 3D position of 36 reflective markers attached to the participants’ lower extremity while they performed a bilateral jump-landing task. The landing task adopted in this study (see below) is a common test to investigate biomechanical landing safety in ACLR athletes [25]. The markers placed bilaterally on the specific anatomic landmarks of the feet, ankles, shanks, knees, thighs, and pelvis to track segments’ motion during each trial. A three-second upright standing static trial was recorded before the landing trials, to align the participant with the laboratory coordinate system and to build subjects’ static reference model for kinematic analyses.

### Jump-landing task

All participants performed a light intensity pedaling on a stationary bicycle for 5-min to warm-up before participating in the jump-landing test. For the jump-landing task, the participants jumped from a 30 cm box and landed at a distance of 50% of body height away from the box (as the landing area) followed by a maximal vertical jump [9]. Each participant performed 2 practice trials, followed by 10 actual test trials. A trial was considered successful if the individual jumped bilaterally from the box, landed in the landing area. Participants could rest at least 1 min between the consecutive trials. Participants wore a pair of standard athletic shoes that has previously been adjusted to place the reflective markers.

### Kinematic data analysis

Ten successful jump-landing tests for each limb were used for further analyses using Visual3D software (C-motion Inc., Kingston, Canada). Data was low pass filtered with a cutoff frequency of 6 Hz followed by identifying the initial contact using the lower leg segment’s vertical velocity. We concentrated on the initial contact of the landing task because previous studies demonstrated that ACL injury/re-injury mostly occurs at the initial contact of landing [8, 26, 27]. That is, the minimum vertical velocity of the lateral malleolus marker [16] was used as the initial contact moment. Moreover, manual inspection was used to verify correct identification and adjustment when required. Cardan angles were used to define the joint angles. The sequence of rotation was x-y-z, based on the recommendations of the International Society of Biomechanics [28]. Hip, knee and ankle kinematics in the three anatomical planes of motion were examined at initial contact, and the average of the 10 landing trials was used for statistical analysis. The positive sign assigned to the hip and knee flexion, adduction, and internal rotation, ankle dorsiflexion, and ankle eversion.

### Statistical analysis

Statistical analysis was completed using IBM SPSS Statistics Version 25.0 (SPSS, Inc., Chicago, IL), and statistical significance was set at *p* < 0.05. Normality of distribution of kinematic data was checked using Shapiro–Wilk normality tests. The limb symmetry indices for the functional, quadriceps strength and questionnaires tests (Table 2), and demographic characteristics (Table 1) were compared between the three groups using one-way analysis of variances (ANOVAs). The kinematic variables were compared between the limbs (involved/uninvolved) and the groups (passed-RTS, failed-RTS and healthy) using nine separate mixed ANOVAs. Post-hoc tests with Bonferroni adjustment were performed to locate the source of significance.

**Table 2:** Return to sport criteria, between groups comparison

	Healthy mean (SD)	Passed- RTS mean (SD)	Failed- RTS mean (SD)	F	Sig.
Quadriceps LSI	100.8 (8.3)	103.3 (17.6)	93.1 (8.1) Failed = 2, Passed = 11	3.169	0.053
Single hop LSI^a^	101.6 (5.2)	98.7 (4.9)	95.0 (7.7) Failed = 1, Passed = 12	**3.467**	**0.041**
Triple hop LSI^a^	102.7 (4.6)	100.5 (2.9)	96.1 (9.4) Failed = 0, Passed = 13	**4.049**	**0.025**
Crossed hop LSI^a, b^	101.1 (4.1)	100.1 (0.3)	93.5 (10.2) Failed = 2, Passed = 11	**4.980**	**0.012**
6-m hop LSI	103.1 (10.6)	101 (7.5)	100.7 (15.1) Failed = 0, Passed = 13	0.188	0.830
Global rating scale	–	93.3 (4.1)	82.5 (10.3) Failed = 10, Passed = 3	**12.589**	**0.001**
KOS-ADL	–	95.1 (4.5)	76.0 (9.3) Failed = 6, Passed = 7	**45.082**	**< 0.001**

*Abbreviations*: *RTS* Return to Sport, *LSI* Limb symmetry index, *KOS-ADL* Knee Outcome Survey Activities of Daily Living Scale, *F* F ration, *Sig* Significance set at *p* < 0.05; ^a^ indicates significant differences between failed and healthy; symbol ^b^ indicates significant differences between failed and passed

## Results

There was no significant difference between the failed-RTS and the passed-RTS groups for the time passed from ACL reconstruction (*p* = 0.73). In addition, there were no significant differences between the groups for age, height, weight and the Tegner score before the injury (*p* > 0.05; Table 1).

The failed-RTS group had lower symmetry index for the single hop (*p* = 0.036), triple hop (*p* = 0.024) and crossed hop (*p* = 0.017) tests compared to the healthy group. However, there were no significant differences between the failed- and passed-RTS groups and the passed-RTS and the healthy group for the hop tests (*p* > 0.05) regarding the limb symmetry index. Moreover, there was no significant difference between the three groups in terms of the quadriceps strength symmetry index (*p* > 0.05). However, participants in the failed-RTS group had lower Tegner score (6 months after ACLR) compared to the passed-RTS group (F = 11.1, *p* = 0.002). The failed-RTS group also obtained lower scores for the global rating knee scale and KOS-ADL compared to the passed-RTS group (Table 2). Table 3 present raw values of each RTS tests.

**Table 3:** Return to sport criteria, raw data

	Failed mean (SD)		Passed mean (SD)		Healthy mean (SD)	
	involved	Non-involved	involved	Non-involved	Non-dominant	dominant
Quadriceps strength (N)	64.3 (14.2)	65.1 (13.5)	66.4 (7.8)	68.1 (11.9)	59.7 (12.3)	61 (13)
Single hop (cm)	167.4 (25)	174.3 (23.58)	189.1 (24.7)	189.5 (22.37)	213.6 (11.56)	211 (18)
Triple hop (cm)	485.5 (67.9)	507 (81.8)	578.4 (89.1)	573.5 (87.6)	603.3 (82.5)	591.3 (77.4)
Crossed hop (cm)	417 (77.8)	441.6 (83.13)	454.3 (88.2)	451.6 (80)	544.3 (63.3)	538 (63.9)
6-m hop(s)	2.4 (0.5)	2.4 (0.6)	2.1 (0.4)	2.2 (0.5)	1.5 (0.3)	1.6 (0.3)
Global rating scale	75 (9.8)	91.2 (10.3)	90.5 (10.1)	96.1 (10.4)	–	–
KOS-ADL	68.3 (9.3)	89.1 (9.8)	91 (8.8)	97 (10.2)	–	–

*Abbreviations*: *N* Newton, *cm* centimeter, *s* second, *SD* standard deviation

### Kinematics

No significant limb-by-group interactions were found for the hip, knee, and ankle 3D kinematics (*p* > 0.05). The main effect of group was significant for hip abduction/adduction (F = 10.541, *p* < 0.001), hip rotation (F = 5.081, *p* = 0.011) and ankle eversion/inversion (F = 11.384, *p* < 0.001). The post-hoc comparisons revealed lower hip abduction angle in both failed (involved limb 10.6 ° ± 4.2, *p* < 0.001) and passed RTS (involved limb 11.6° ± 3.1, *p* = 0.006) groups compared to the healthy group (non-dominant limb 16.6° ± 3.7). However, hip abduction was not different between the failed- and passed-RTS groups (*p* > 0.05). With regard to hip transverse plane kinematics, post-hoc test found lower hip external rotation in the failed-RTS group (involved limb 12.7° ± 6.1) compared to the healthy group (non-dominant limb 15.7° ± 8.3, *p* = 0.01), but no significant differences between the failed- and passed-RTS groups (*p* = 0.152), and also between the passed-RTS and healthy groups (*p* = 0.923).

There were no significant differences between the groups in knee kinematics (*p* > 0.05). Ankle inversion in the failed-RTS (involved limb 10.2° ± 5.1) was significantly lower than both the passed-RTS (involved limb 12.1° ± 4.8, *p* = 0.05) and the healthy (non-dominant limb 22.1° ± 8.2, *p* < 0.001) groups. Finally, there was no significant difference between the passed-RTS and the healthy groups for ankle inversion/eversion (*p* = 0.117).

**Fig. 1:**
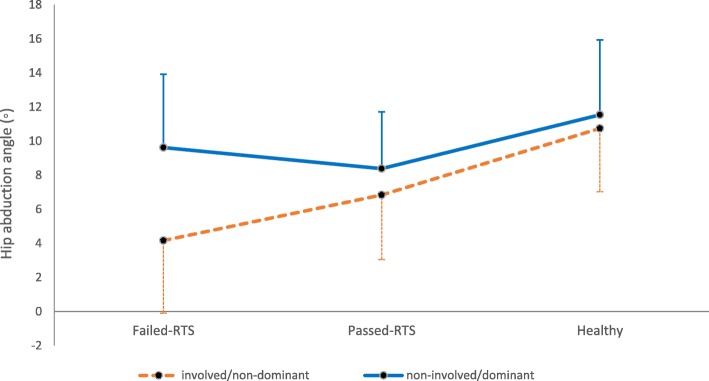
limb comparison for hip abduction in Failed-RTS, Passed-RTS and Healthy group

The main effect of limb was only significant for hip abduction/adduction (F = 9.383, *p* = 0.006). The involved limb of failed-RTS had lower hip abduction compared to the non-involved limb (mean difference = 5.45° ± 6). The passed-RTS group and the healthy athletes had relatively similar hip abduction/adduction (Table 4 and Fig. 1).

**Table 4:** The mean (standard deviation) of lower extremity angles (°) at the initial contact of landing

	Hip		Knee				Ankle		
	Flex	Add	Int Rot	Flex	Add	Int Rot	DorsiFlex	Eversion	Int Rot
Healthy									
Non-dominant	37.8 (8.3)	−16.6 (3.7)	−15.7 (8.3)	68.4 (6.2)	−4.5 (6.4)	− 11.5 (8.4)	11.1 (4)	−22.1 (8.2)	−9.1 (7.5)
Dominant	37.6 (9.1)	−17.8 (4.4)	−19.3 (6.4)	64.4 (7.1)	−4.5 (9.1)	−22.6 (8.3)	8.4 (5.1)	−16.4 (5.2)	−6.8 (6.2)
Passed-RTS									
Involved	44.3 (7.1)	−11.6 (3.1)	−15.1 (6.1)	63.2 (7.6)	−0.8 (4.1)	−13.5 (7.1)	12.8 (3.3)	−12.1 (4.8)	−9.5 (6.2)
Non-involved	43.2 (6.7)	−14.6 (3.1)	−19.1 (5.3)	66.1 (7.5)	−4.5 (5.1)	−15.5 (5.1)	11.1 (3.8)	−11.7 (4.1)	−2.8 (3.3)
Failed-RTS									
Involved	34.5 (9.8)	−10.6 (4.2)	−12.7 (6.1)	62.4 (6.3)	−18.1 (8.3)	−17.1 (9.1)	12.8 (3.8)	−10.2 (5.1)	−7.2 (7.3)
Non-involved	40.4 (10.6)	−17.6 (4.3)	−17.5 (6.8)	67.1 (6.4)	−8.5 (6.3)	−15.6 (7.1)	13.1 (3.4)	−7.2 (4.8)	−13.2 (7.1)

*Abbreviations*: *Flx* flexion, *Ext* extension, *Abd* abduction, *Add* adduction, *Int/Ext Rot* Internal/External Rotation

For the ankle joint: Flx: Dorsi-flexion, Ext: Plantar Flexion, Abd: Eversion, Add: Inversion

## Discussion

The aim of this study was to compare lower limb kinematics of 3 groups of athletes including two ACL reconstructed (passed- and failed-RTS criteria) and a healthy group. In support of our hypothesis, we found significant differences in the hip and ankle kinematics between the passed-RTS, failed-RTS and healthy athletes. Our findings revealed reduced hip abduction in athletes who passed RTS compared to the healthy athletes despite an acceptable functional status in RTS. However, there was no significant difference in hip abduction angle between the passed- and failed-RTS groups although having different RTS criteria. This implies that RTS criteria cannot identify improper lower extremity kinematics in a landing task which could signify the cause of re-injury in passed-RTS ACLR athletes. Our results also demonstrated a significant difference between the failed- and passed RTS groups in ankle inversion/eversion angle.

Quadriceps strength of failed, pass and healthy athletes was similar for both involved and non-involved limb (dominant and non-dominant for healthy athletes). For all hop tests, healthy athletes obtained greater score compared to the failed and passed groups. Moreover, pattern of raw data and LSI (Tables 2 and 3) of each criterion was similar. This shows that not only are those in the Passed-RTS group more symmetrical than the failed group that they also scored better on every outcome (farther jump distances, higher strength, better self-reported outcomes). In our study, number of athletes who failed two self-report questionnaires were higher that those who failed the hop tests. This may indicate the role of psychological factors for readiness to return to sport [29, 30]. Based on Table 2, a maximum of only 3 people failed the RTS criteria for any hop test. While group analyses showed that the people in the Failed-RTS group were significantly different than controls for the single, triple and crossed hops. Similar finding was also reported by Ebert et al. (2018) [31]. This might show that the hop tests have low sensitivity for discriminate ACLR athlete who may be at risk of re-injury.

### Comparison of the passed-RTS and healthy group

In the passed-RTS group, despite appropriate improvement in the muscle strength and functional performance (LSI ≥ 90%), lower hip abduction angle was found compared to the healthy group. This result is consistent with previous studies that reported a tendency toward hip adduction during walking and landing in individuals with ACLR who returned to play [25, 32]. Research suggest an association between ACL injury and excessive hip adduction and internal rotation during multiplanar movement, e.g., landing [8, 33]. Thus, reduced hip abduction and external rotation seen in ACLR athletes who passed RTS criteria may indicate that current strength and function-based RTS criteria is not sufficient to identify athletes at risk of ACL re-injury and highlights the need for lower extremity kinematic analysis together with current RTS criteria. Altered hip kinematics in athletes who passed RTS may also suggest the need for more attention to the strengthening of the hip and core stabilizers, and neuromuscular training following ACLR as positive effects of hip strengthening and neuromuscular training on changing frontal plane hip motion reported in previous studies [34–36].

### Comparison the failed- and passed-RTS group

The failed- and passed-RTS groups were only statistically different in the ankle inversion, with no significant differences in the hip and knee kinematics. Consistent with our finding, Chang et al. (2019) reported no significant difference in knee kinematics between female athletes who pass and fail the RTS criteria. The similar hip and knee kinematics in the two groups, even with different RTS scores, could indicate that functional or strength asymmetry is not associated with the kinematic characteristics of lower extremity in the ACLR athletes. This is in agreement with the results of a recent study by Xergia et al. (2015) who found no significant correlation between functional asymmetry assessed by LSI for the hop test and lower extremity kinematics during landing [37]. However, similar hip and knee kinematics of failed- and passed-RTS groups in our study is contrary to the finding of Di Stasi et al. (2013) who found different hip and knee kinematics in the passed- and failed-RTS groups during treadmill walking [5]. This controversy may be due to the differences in the task evaluated, as it has been shown that the kinematics of gait and landing tasks are not associated [38].

Similar lower extremity kinematics in failed- and passed-RTS groups indicates that passed RTS athletes still have biomechanical risk factors for ACL re-injury similar to the failed group despite passing the return to sport criteria. Therefore, kinematic analysis together with current RTS may provide more detailed information for increased safety to return to sport decision making.

In our study, participant in the failed-RTS group landed with less ankle inversion when compared with the passed-RTS group. Because of linked motion between lower extremity joints during close kinetic chain tasks such as landing, changes in the ankle frontal plane motion may impact hip and knee joint kinematics [39, 40] and may increase the risk of ACLR injury/re-injury [41]. Previous studies reported that Change in ankle motion during landing may contribute to Transverse plane hip and frontal plane knee positioning when contacting the ground, which are known to increase the risk of lower extremity injury [42, 43].

### Comparison of the failed-RTS and healthy group

Hip abduction (mean diff = 4.5°), hip external rotation (mean diff = 5.6°), and ankle inversion (mean diff = 7.8°) were significantly lower in the failed-RTS group compared to the healthy group. Previous studies also reported significant differences between the ACLR and healthy groups in peak hip flexion [17, 24], hip abduction/adduction [25] and frontal plane knee motion [44]. Decreased hip abduction, external rotation and ankle inversion may increase tendency toward dynamic valgus phenomena in the initial phase of landing which is an important risk factor to ACL re-injury [8]. Paterno el al. (2010) in a prospective study also reported that excessive inward motion of lower extremity in frontal plane during landing results in three times increased risk of ACL re-injury following ACL reconstruction [8]. These kinematic changes in the lower extremity joints in the failed RTS group, even several months after the reconstruction surgery and rehabilitation, may further highlight the need for more attention to movement education/training and neuro-biomechanics of motion during ACLR rehabilitation.

### Limb comparison

We found lower hip abduction angle in the ACL reconstructed limb compared to the intact limb in the failed-RTS athletes. This may explain the higher rate of re-injury in the ACL reconstructed limb in individuals with a history of unilateral ACLR. That is, increased hip adduction, which can lead to a valgus knee position, is known as an important risk factor that can predict the risk of ACL re-injury [8]. Adam et al. (2018), in a meta-analysis, also showed that individuals with ACLR land with increased hip adduction in the involved limb compared to the intact limb [25].

### Study limitation

One limitation of our study is that we did not evaluate hip muscles strength. This was because the focus of this study was to investigate the association between RTS criteria and lower extremity kinematics. Several studies, however, reported a relationship between hip strength and lower extremity kinematics during challenging activities such as landing [45, 46], step decent [47] and hopping [48]. Future studies should consider hip muscle strength while comparing lower extremity kinematics in the ACLR knee. Moreover, this study included only male participants which limit generalizability of our outcomes to females with ACLR. This was mainly due to the low rate of female ACLR athletes in our available population. Also considering kinetic data in conjunct with kinematics in the future studies can improve our knowledge about landing biomechanics as a suggested task to evaluation of athlete readiness to return to sport after ACLR and its relationship with current RTS criteria. Finally, Current RTS testing is performed under pre-planned/anticipated circumstances. There is a plethora of studies investigating the effects of unplanned/unanticipated jump landings/cuttings on lower limb biomechanics [49–51]. As team sport athletes interacting in a variable and unforeseen environment, such tests may better mimic the realistic affordances (increase ecological validity).

## Conclusion

Our finding indicated reduced hip abduction during landing in athletes who released to return to sport despite nearly normal functional and muscle strength scores. Reduced hip abduction during a complex multi-planar movement such as jump-landing is a risk factor for ACL re-injury. Most of current RTS criteria are mainly based on muscle strength and functional evaluation without consideration of alteration in lower extremity kinematics. Findings of this study support our hypothesis that current RTS criteria may not be sufficient to differentiate athletes at high risk of ACL re-injury. Kinematic analysis in conjunction with current RTS criteria can provide additional insight into return to sports decision making.

## Data Availability

The datasets used and/or analyzed during the current study are available from the corresponding author on reasonable request.

## Abbreviations

ACL: Anterior cruciate ligament
ACLR: Anterior cruciate ligament reconstruction
GRS: Global rating scale
KOS-ADL: Knee outcome survey scale-activity daily living
LSI: Limb symmetry index
RTS: Return to sport
